# Learning-based prediction of gestational age from ultrasound images of the fetal brain

**DOI:** 10.1016/j.media.2014.12.006

**Published:** 2015-04

**Authors:** Ana I.L. Namburete, Richard V. Stebbing, Bryn Kemp, Mohammad Yaqub, Aris T. Papageorghiou, J. Alison Noble

**Affiliations:** a Institute of Biomedical Engineering, Department of Engineering Science, University of Oxford, Oxford, United Kingdom; b Nuffield Department of Obstetrics and Gynaecology, University of Oxford, Oxford, United Kingdom

**Keywords:** Fetal brain ultrasound, Gestational age, Regression forest, Surface parametrization, Brain development

## Abstract

We propose an automated framework for predicting gestational age (GA) and neurodevelopmental maturation of a fetus based on 3D ultrasound (US) brain image appearance. Our method capitalizes on age-related sonographic image patterns in conjunction with clinical measurements to develop, for the first time, a predictive age model which improves on the GA-prediction potential of US images. The framework benefits from a manifold surface representation of the fetal head which delineates the inner skull boundary and serves as a common coordinate system based on cranial position. This allows for fast and efficient sampling of anatomically-corresponding brain regions to achieve like-for-like structural comparison of different developmental stages. We develop bespoke features which capture neurosonographic patterns in 3D images, and using a regression forest classifier, we characterize structural brain development both spatially and temporally to capture the natural variation existing in a healthy population (N=447) over an age range of active brain maturation (18–34 weeks).

On a routine clinical dataset (N=187) our age prediction results strongly correlate with true GA (r=0.98,accurate within±6.10days), confirming the link between maturational progression and neurosonographic activity observable across gestation. Our model also outperforms current clinical methods by ±4.57 days in the third trimester—a period complicated by biological variations in the fetal population. Through feature selection, the model successfully identified the most age-discriminating anatomies over this age range as being the Sylvian fissure, cingulate, and callosal sulci.

## Highlights

• We present a model to predict gestational age from 3D fetal brain ultrasound images.
• A feature-based model characterizes spatial and temporal brain development.
• We capitalize on sonographic image patterns and clinical measures to predict age.
• Use of clinical measurements and neuroimage information improves age predictions.
• We identify the most age-discriminating brain anatomies in early brain development.

## Graphical abstract

**Figure:**
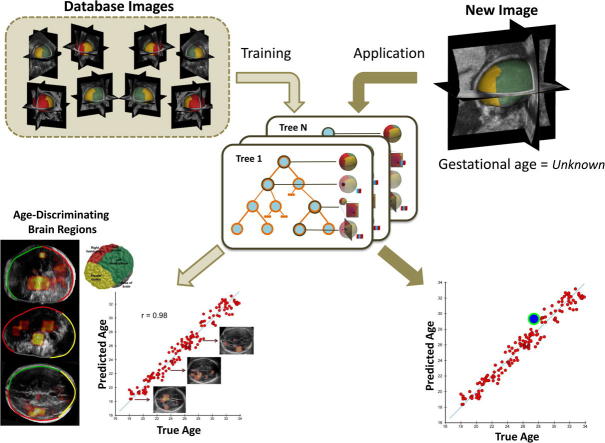

## 1 Introduction

Accurate gestational age (GA) estimation forms an integral part of obstetric prenatal care. It defines the estimated date of delivery (EDD), and can influence the success or safety of a clinical intervention. Moreover, it is essential for the screening of fetal abnormalities. The anomaly scan, which is routinely offered in the early second trimester, forms the legal basis for time-critical care decisions as it enables expectant parents to make informed reproductive decisions about their unborn child (e.g. termination of pregnancy, intrauterine therapy or intervention) (National Collaborating Centre for Women’s and Children’s Health, 2008).

Traditional approaches to GA estimation include (a) menstrual dating, which makes use of the first day of the last menstrual period (LMP) as a reference point for the EDD and (b) extraction of diameter and circumference measurements from 2D ultrasound (US) images of the fetal cranium, abdomen, and femur (ISUOG, 2007). These measurements are regressed to population-based dating charts to estimate age and assess normality of fetal growth (Loughna et al., 2009). However, beyond 24 post-menstrual weeks, measurement accuracy is dependent on operator expertise and compromised by increasing biological variation, inconsistencies in skull size approximation, and subjectivity in 2D diagnostic plane finding, all contributing to age approximation errors (Bottomley and Bourne, 2009). As pregnancy advances and biological variation amongst normal fetuses increases, the range of values of each biometric measurement associated with a specific GA also increases and so equations based upon size become less accurate. In practice, this means that whilst the predictive error at 22 weeks’ GA (±10 days, Altman and Chitty (1997)) is considered acceptable in the majority of clinical settings, the predictive error at 28–42 weeks (±18 days) is considered to offer little clinical value (Hadlock et al., 1983).

Pregnancy dating becomes particularly important in low-income settings where pregnant women typically attend for obstetric care late in pregnancy, when menstrual history is unavailable or unreliable. In the absence of clinically-useful LMP information, US measurements provide the most accurate estimation of GA (Geirsson, 1991). However, in the third trimester of pregnancy, even US-based dating may produce estimation errors up to ±3 weeks (Hadlock et al., 1984; Altman and Chitty, 1997). Thus, in a setting where screening occurs in the second and third trimesters, the error margins yielded by current methods render them as not clinically useful, potentiating the need for alternative techniques for estimating GA.

Post-mortem neuroanatomical studies have observed that during early development the fetal brain undergoes dramatic structural changes and have established a spatiotemporal timetable which characterises normal brain development (Chi et al., 1977; Dorovini-Zis and Dolman, 1977). Specifically, the fetal brain surface, or *cortex*, rapidly transitions from a relatively smooth agyric surface in the early second trimester to progressively bearing more indentations or folds (*gyrification*) over the course of pregnancy until it resembles the adult brain at birth. Deviations from this pattern have been indicative of cortical malformations as a result of defective neuronal migration, as is the case of lissencephaly which occurs when gyrification is reduced or stunted. Depending on severity, cortical malformation may result in adverse outcomes ranging from developmental delays and retardation to infant mortality (Ghai et al., 2006). This, in turn, is suggestive of a direct link between healthy gyrification and chronological age. These findings raise the question whether changes in brain morphology could be used as a robust indicator of GA and developmental normality in clinical practice.

### 1.1 Related work

To date, several methods have been developed to automatically map neuroanatomical structure from MR image data to neonatal or adult age. Using voxel-based morphometry or shape analysis to capture tissue growth (Good et al., 2001; Gholipour et al., 2012), tensor analysis to characterize regional growth patterns (Thompson et al., 2000), or discriminative classifiers to capture characteristics of the developing or ageing brain (Franke et al., 2012; Sabuncu and Van Leemput, 2012; Toews et al., 2012), a clear link between anatomical changes and cerebral progression (or regression) has been demonstrated. With the advent of image preprocessing methods such as slice-to-volume reconstruction and image mosaicing (Jiang et al., 2007; Rousseau et al., 2006), and super-resolution techniques (Gholipour et al., 2010; Kim et al., 2010; Rousseau et al., 2010), 3D MR images with high signal-to-noise ratio and improved spatial resolution are now available and have stimulated studies of fetal (Caldairou et al., 2011; Gholipour et al., 2012; Habas et al., 2010; Habas et al., 2012; Jacob et al., 2011; Rajagopalan et al., 2011; Scott et al., 2011; Scott et al., 2013; Serag et al., 2012; Dittrich et al., 2014; Wright et al., 2014) and neonatal (Kuklisova-Murgasova et al., 2011; Serag et al., 2012) brain development from MR images. However, these techniques are tailored for the challenges affecting MR images and may not be appropriate for application in neurosonography, which continues to be the modality of choice in routine clinical care.

In the clinical literature, the age-related changes in echogeneity of fetal brain structures have been well-described. The timing of emergence of cortical sulci has been observed in US images and described as following a predefined spatiotemporal timetable (Bernard et al., 1988; Monteagudo and Timor-Tritsch, 1997; Toi et al., 2004; Cohen-Sacher et al., 2006; Pistorius et al., 2010), in agreement with MR and post-mortem neuroanatomical findings. In particular, the process of cortical maturation observable in US images of the fetal brain has been detailed by means of simple subjective scoring techniques to define the appearance of sulci and gyri beyond 20 gestational weeks (GW) (Quarello et al., 2008; Pistorius et al., 2010).

Unlike MR images, US images are complicated by intensity artefacts such as signal attenuation, acoustic shadows, and occlusion due to cranial calcification. US probe placement also generates reverberation caused by multiple reflections of the US beam on the fetal skull and other maternal tissues. These factors can affect the visibility of key anatomical landmarks necessary for image registration—the primordial step in image-based brain analysis. However, given that cranial calcification and fusion progress with GA (Malas and Sulak, 2000), the complex image patterns generated by these artefacts may be used along with structural image features to inform on developmental maturation.

Our work is the first to exploit age-related sonographic activity to predict GA and hence neurodevelopmental maturation from US images. We present bespoke appearance-based features designed to capture these age-specific sonographic patterns and use them to develop a model which automatically maps them to GA and hence neurodevelopmental maturation. Learning-based approaches are well-suited for this task due to their ability to take high-dimensional data (i.e. longitudinal images producing 5000+ features representing image appearance at different ages) and establish a compact representation of fetal brain development. In the literature, relevance vector machines (RVM) (Franke et al., 2010) and relevance voxel machines (RVoxM) (Sabuncu and Van Leemput, 2012) have demonstrated the feasibility of learning a mapping between image-based biomarkers and pathologies in adult brains. More recently, Konukoglu et al. (2013) applied neighbourhood approximation forests (NAF) to estimate adult age. While the work of Konukoglu et al. (2013) also presents a forest-based method for predicting age from brain images, their approach relies on accurate alignment and registration of anatomical landmarks, which remains a challenge in US images of the brain. Unlike MR images, the appearance of anatomies in an US image varies with the relative position of the brain with respect to the probe, which results in acoustic shadows, occluded anatomical features, and reverberation artifacts (Kuklisova-Murgasova et al., 2013). Consequently, approaches requiring images of similar intensity appearance and one-to-one inter-subject anatomical correspondence are not, at present, directly applicable to a study of US images of the brain; a local feature-based approach is more appropriate (e.g. Toews et al., 2010).

The advantages of employing decision forests for such tasks are their built-in automatic feature selection, which allows for identification of salient and age-discriminating image features, and their generalizability to images from different age groups and acquisitions. Thus, decision forests are appropriate for our work in which we seek to identify the structures which are informative for GA decision-making, and aim to apply the model to images from patients at different developmental stages.

### 1.2 The proposed method

**Fig. 1:**
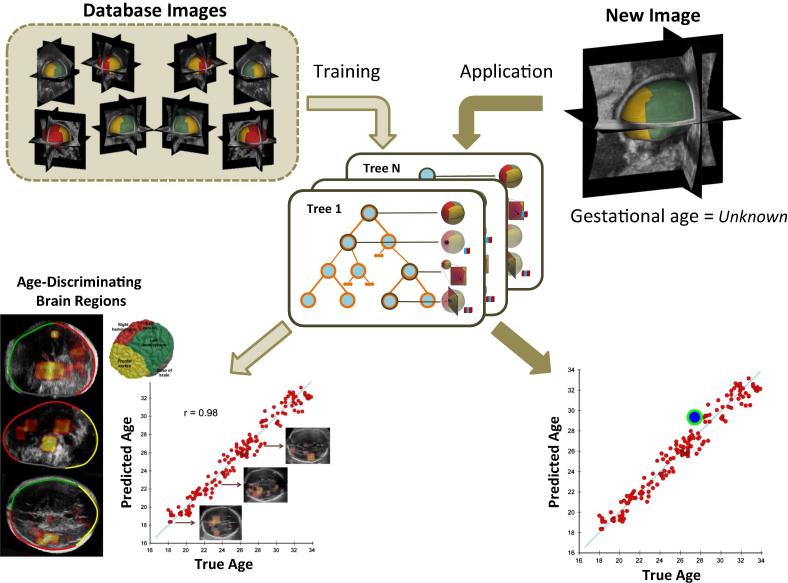
Schematic flow chart of probabilistic age estimation model. Given a set of images in the database, the machine learning model will be trained to learn age-specific ultrasound image appearance and model a mapping between sonographic activity and age. When given a new test image taken from a fetus of unknown gestational age, the model can be applied to estimate age and possibly determine clinical outcomes.

We propose a feature-based model for characterizing neuroanatomical appearance both spatially and temporally, capturing the natural variation existing in a healthy fetal population over a period of active brain maturation: 18^+0^ to 33^+6^ GW (weeks^+days^). Specifically, we present an automated machine learning-based predictive model to learn the pattern of fetal brain changes through dynamic features observable in multiple subject images and apply it to demonstrate successful age estimation from a single unseen scan. Our proposed model comprises of two steps: (i) 3D parametrization of the fetal skull and (ii) feature extraction for learning age-related sonographic patterns from 3D volumes, resulting in the development of an age-predictive model. The model can then be applied to a new 3D US volume to automatically predict the GA of the fetus from which the volumetric image was collected. Fig. 1 presents an overview of the GA estimation framework.

To tackle the challenges posed by fetal neurosonography, we proposed the use of a 3D continuous B-spline surface to model and parametrize the fetal skull (Namburete et al., 2013). Cranial parametrization allows for fast and efficient sampling of anatomically-corresponding brain regions to achieve like-for-like structural comparison of different developmental stages—voiding the need for segmentation or registration of intracranial structures (Namburete et al., 2014b). The lack of direct registration encourages preservation of age-specific sonographic patterns and image differences, which may be indicative of GA. The methodology for cranial parametrization was first described and validated in our previous conference papers (Namburete et al., 2013; Namburete et al., 2014a), and Section 2.1 describes the process in detail.

It has been demonstrated that the parametric surface is suitable for feature extraction in a supervised age-predictive framework based on regression forests to predict the GA of healthy and routine clinical fetal populations (i.e. containing growth-restricted fetuses) in the second trimester (18^+0^ to 28^+6^ GW) (Namburete et al., 2014b). That approach used age-related changes in US image intensity, distance of near-neighbour points on the cranial surface, and inner head circumference features for the age regression and prediction. We demonstrated that our model successfully estimated GA on a set of 130 healthy fetuses (error of ±3.8 days), outperforming the current best clinical method. Through the feature selection process, the model also identified the Sylvian fissure as a critical age-discriminating region during this age range.

In this paper, we further extend the age range of the model to function in the second and, importantly, third trimesters (i.e. 18^+0^ to 33^+6^ GW) to make the method clinically useful. We describe, in detail, the features used in the regression forest model for age prediction (§2.3), and through further validation experiments, we demonstrate the ability of the model to accurately predict GA from 3D US images (§4). We also provide new insight into the functionality and behaviour of the age-predictive model, and examine the effect of feature inclusion on prediction accuracy. Finally, we show, for the first time, that the proposed age estimation framework is capable of identifying age-discriminating regions in the fetal brain, which has never before been demonstrated on clinical US images.

## 2 Methods

### 2.1 Cranial parametrization

This section describes the 3D cranial parametrization method in preparation for US image analysis of the fetal brain. Throughout, matrices are denoted by boldface uppercase letters (X), vectors by boldface lowercase letters (x), and column-vectors extracted from matrices are denoted by indexed boldface letters, such that $x_{i}$ is the *i*-th column of matrix X. The *j*-th element of vector x is denoted by $x_{j}$.

Cranial parametrization is performed by a semi-automated framework that fits a continuous 3D parametric surface into the skull in each US image. The input to the framework is a 3D US image to which the anatomically annotated surface is manually aligned. Though providing coarse inter-subject alignment, the surface does not attempt to achieve one-to-one correspondence between brain regions of different subjects but rather ensures that, when present and visible, the anatomical regions are approximately aligned for feature extraction. The aligned surface serves as the initialization to an energy minimization algorithm which seeks to deform the surface until it finds the best fit to the inner skull boundaries in the image. During deformation, the surface geometry changes to adhere to the cranial edges, but the topology of the surface points is preserved.

The remainder of this section describes the basic cranial parameterization framework which is further detailed in Namburete et al. (2013) and validated in Namburete et al. (2014a). The surface fitting framework is inspired by “Snakes” (Kass et al., 1988) and Doo-Sabin surface fitting (Orderud et al., 2007) but differs in three distinct ways. First, surface “fit” is defined as the squared distance between positions on the model surface and a *subset* of detected edge positions in the image. In comparison, “Snakes” define model fit directly against the image and in Orderud et al. (2007), edge positions are detected at each step when updating the model surface. Second, the coordinates of positions on the surface model—which are used to define the surface fit—are optimized jointly with the surface geometry. In contrast, in “Snakes” and Orderud et al. (2007) the coordinates are fixed. The joint optimization is inspired by the works of Cashman and Fitzgibbon (2013) and Marinov and Kobbelt (2004), and although it is more complex, it has two key advantages: (a) selected edge positions are *not* restricted to be perpendicular to the surface model as in Orderud et al. (2007) and (b) surface folding is discouraged. Third, in Orderud et al. (2007), edge positions are detected and selected *independently* for each position on the surface model. The assumption of independence simplifies edge detection and selection, but does not encourage a *coherent* selection of edge positions across the surface. To remedy this, pairwise terms are included here so that the edge positions selected for neighbouring points on the surface model are encouraged to be close. This has been done previously for *open* model contours in 2D for which the discrete optimization problem can be solved exactly using dynamic programming (Behiels et al., 2002). For a closed surface in 3D, such as the one presented here, the resulting discrete optimization problem is more complex.

#### 2.1.1 Surface model

**Fig. 2:**
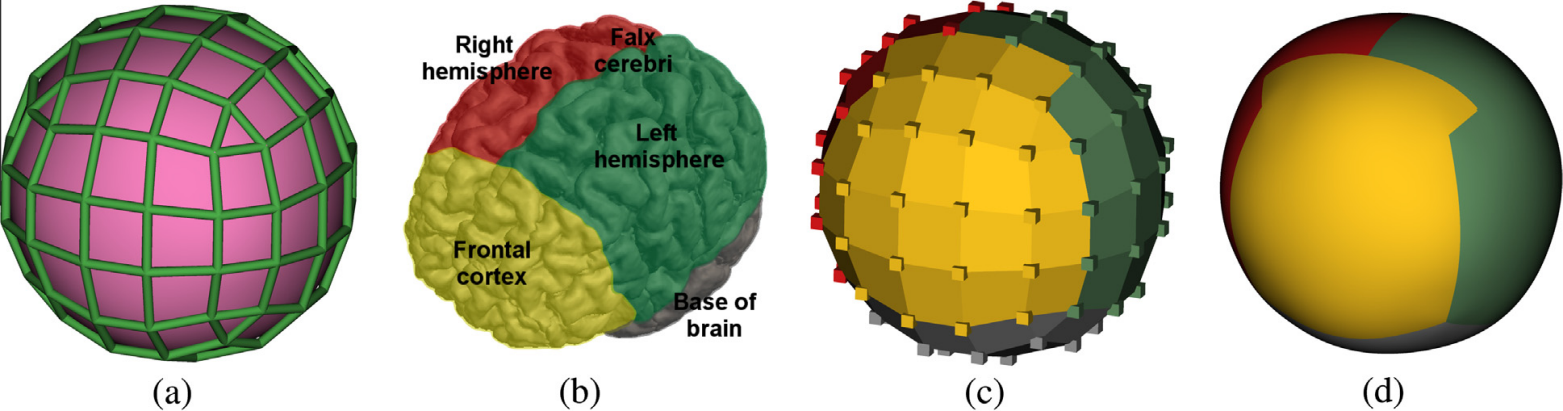
Surface model: (a) The control mesh and control vertices (X, green) defines its underlying surface (pink). (b) Given four coarse anatomical regions, (c) the control vertices and faces are colour-annotated with the anatomical regions with which they ought to align in the image during surface initialization. (d) These annotations are propagated down to the underlying cranial surface. (For interpretation of the references to colour in this figure legend, the reader is referred to the web version of this article.)

We generated a bespoke spherical biquadratic B-spline surface (Piegl and Tiller, 1997) which can be aligned to anatomical regions within the brain. The control mesh T explicitly represents the underlying cranial surface (Fig. 2(a), in pink), and its geometry is specified by $N_{X}$ control vertices, $X\in R^{3\times N_{X}}$ (Fig. 2(a), in green). Each point p on the cranial surface is parametrized by a surface coordinate u∈Ω in the surface domain $\Omega \subset R^{2}$ through a mapping function $M:\Omega \times R^{3\times N_{X}}\to R^{3}$ such that p=M(u,X). Similarly, the surface normal at u is defined by $n=M_{\phi }(u\text{,}X)$, with mapping function $M_{\phi }:\Omega \times R^{3\times N_{X}}\to R^{3}$.

#### 2.1.2 Surface Initialization

**Fig. 3:**
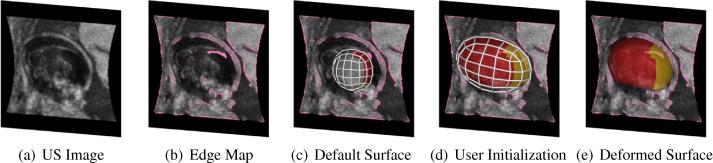
Surface initialization and deformation Sagittal view of (a) an example US image of the fetal head with (b) the edge map enhancing ridge-like features, (c) default annotated surface X, (d) user-initialized surface $X^{0}$, and (e) 3D rendering of the final deformed surface superimposed on the US image.

To initialize the surface control vertices X to $X^{0}$, the user rigidly aligns the default spherical surface to the imaged skull/brain using a multi-view graphical user interface (GUI) programmed using VTK.^1^ As shown in Fig. 3(d), initialization involves displacing the surface to roughly align with the centre of the brain, rotating and anisotropically scaling the surface such that it approximates the cranial dimensions and aligns anatomical regions to surface annotations. This manual interaction is a simple process which takes approximately 1–2 min per 3D image when performed by a non-clinical expert (Namburete et al., 2013).

To facilitate manual initialization, the faces on the control mesh were colour-annotated with the anatomical regions with which they ought to align in the US image (Fig. 2(b) and (c)). The colour annotations were based on a coarse parcellation of the brain, including a minimal number of landmarks observable in US images which would allow for an approximate anatomical alignment, despite the complications caused by partial occlusions. These anatomical regions include the right and left cerebral hemispheres, the frontal cortex, and the falx cerebri (structure separating the cerebral hemispheres) (Fig. 2(b)). Rigid image alignment using these global landmarks provides information about approximate brain size, fetal head pose and orientation (e.g. cephalic or breech). The colour annotations on the control faces of T are propagated down to define colouring of the underlying surface (Fig. 2(d)).

#### 2.1.3 Surface deformation

Determining the ideal deformation for the surface model to fit to the imaged cranial boundary first requires selection of relevant boundary candidates in the US image. For the purposes of brain analysis, the ideal voxel candidates lie in the interior skull boundary which we generated using a standard US edge detection technique: Feature Asymmetry (FA) (Kovesi, 1996). The FA image retains important structural information, is contrast-invariant, and allows edge features to be obtained at different scales. In the task of cranial boundary detection, we are interested in the junction between the inner skull boundary and the intracranial soft tissue which behaves like step-edges or ridge-like structures, so an isotropic log-Gabor filter is appropriate (Boukerroui et al., 2004). Non-maximum suppression was then applied to thin the edges in the FA image.

Given an original US image (Fig. 3(a)), candidate interior skull positions $C\in R^{3\times N_{C}}$ and normals $\Phi \in R^{3\times N_{C}}$ are defined from the corresponding FA edge image (Fig. 3(b)). Thus, given control vertices X, and a matrix U of $N_{U}$ surface points, the energy function defining the fit of the surface to a selection of edge image boundary candidates, $q\in N^{N_{U}}$, is given by:

(1) $$E(q\text{,}U\text{,}X)=\overset{N_{U}}{\underset{i=1}{\sum }}E_{\text{unary}}(q_{i}\text{,}u_{i}\text{,}X)+\lambda_{2}E_{\text{pairwise}}(q)+\lambda_{3}E_{\text{user}}(X)+\lambda_{4}E_{\text{reg}}(X)$$

where $\lambda_{2}\text{,}\lambda_{3}\text{,}\lambda_{4}$ influence the weight of each term.

Specifically, $E_{\text{unary}}$ quantifies the mismatch between each surface point $u_{i}$ and its corresponding boundary point at $q_{i}$ in terms of position and orientation.

(2) $$E_{\text{unary}}(q_{i}\text{,}u_{i}\text{,}X)={\left\|c_{q_{i}}-M(u_{i}\text{,}X)\right\|}^{2}+\lambda_{1}{\left\|\phi_{q_{i}}-M_{\phi }(u_{i}\text{,}X)\right\|}^{2}$$

where $\lambda_{1}$ penalizes the orientation of the vectors between the surface and boundary points. Note that boundary candidates are not assumed to be perpendicular to the surface model.

Realizing that by virtue of anatomical structure boundary points are spatially correlated, so the $E_{\text{pairwise}}$ term models the fact that neighbouring surface points should also prefer boundary points which are spatially coherent.

(3) $$E_{\text{pairwise}}(q)=\underset{(i\text{,}j)\in N}{\sum }{\left\|c_{q_{i}}-c_{q_{j}}\right\|}^{2}$$

where N is the set of edges over the surface points.

Given that the surface model is manually initialized by an individual who provides approximate anatomical surface orientation and head size, it is desirable that the surface deformation does not deviate too far from its original surface placement. Consequently, $E_{\text{user}}$ encourages minimum deformation from the user initialization, and also removes the problem of finding multiple local minima that may arise from the geometric symmetry of the near-ellipsoidal shape of the fetal skull.

(4) $$E_{\text{user}}(X)=\overset{N_{X}}{\underset{i=1}{\sum }}{\left\|x_{i}-x_{i}^{0}\right\|}^{2}$$

Finally, the regularization term $E_{\text{reg}}$ encourages a smooth cranial surface by penalizing large displacements between the surface control vertices.

(5) $$E_{\text{reg}}(X)=\underset{(i\text{,}j)\in T}{\sum }{\left\|x_{i}-x_{j}\right\|}^{2}$$

While Eqs. (3) and (5) appear similar, they serve different purposes: $E_{\text{pairwise}}$ regularizes the selection of boundary points and is a function over the vector of *discrete* boundary point indices; $E_{\text{reg}}$ regularizes the surface model and is a function over the matrix of *continuous* model control point positions.

Eq. (1) is minimized through a series of alternating discrete and continuous optimization steps. By taking the user initialization of X to $X^{0}$ and generating U to a regular sampling of *Ω*, we use belief propagation with a subset of edges in $E_{\text{pairwise}}$ to solve an approximate q which is then refined using Quadratic Pseudo-Boolean Optimisation (QPBO) (Kolmogorov and Rother, 2007). Next, given q, Eq. (1) is minimized *jointly* with respect to X and U using the Levenberg–Marquardt algorithm (Gill et al., 1981).

Importantly, as a result of the convexity of the surface, $E_{\text{reg}}$, and due to the *joint* optimisation of X and U^2^, surface self-intersection and stretching is strongly discouraged and was not seen in practice. The resulting surface is an anatomically realistic representation of a ‘complete’ fetal skull (i.e. filling the gaps of fontanelles).

### 2.2 Extracting features from the cranial domain

For the task of predicting GA, and hence maturation, from brain images, the features were designed with the objective of extracting morphological changes guided by the findings of post-mortem neuroanatomical studies of early brain development (Chi et al., 1977; Dorovini-Zis and Dolman, 1977). Specifically, the second and third trimesters are marked by (a) increasing cortical complexity with the emergence and developmental progression of sulci and gyri on the fetal brain surface (Pistorius et al., 2010; Toi et al., 2004; Wright et al., 2014); (b) an increase in overall brain size and volume (Chang et al., 2000; Zhang et al., 2013); and (c) changes in brain surface curvature in different cortical regions (Aljabar et al., 2011; Clouchoux et al., 2012; Habas et al., 2012). We designed three features banks, each capitalising on this prior knowledge about anatomical maturation, to be used in our machine learning framework to develop a link between cerebral progression and GA.

Typical approaches to mapping anatomical structure from image data to age have relied on accurate inter-subject segmentation and registration of brain images (Good et al., 2001; Thompson et al., 2000; Franke et al., 2012; Sabuncu and Van Leemput, 2012) or atlas creation (Dittrich et al., 2014), which remains a challenge in US images of the fetal brain. In particular, when working with US images of fetal brain anatomy, one cannot rely on the assumption that inter-subject alignment is capable of achieving one-to-one anatomical correspondence in subjects of different ages. Given that our data-driven model employs the image representation to discover distinctive anatomical patterns related to ageing, it may be likened to Feature-Based Morphometry (FBM) (Toews et al., 2010) as it aims at quantifying feature variability based on cortical appearance, geometry, and occurrence statistics at different GAs. The features capture local patterns of anatomical development, and the parametrized surface model fulfils the role of common coordinate space, voiding the need for a global brain atlas. The features are thus sampled with respect to the parametrized surface model, ensuring inter-subject anatomical consistency in the image regions sampled for feature extraction (Namburete et al., 2014a).

#### 2.2.1 Feature design

3D images contain a large amount of information and possibly several neighbouring image patches containing similar information. Reducing the number of surface/image ‘points’ included in the search space reduces redundancy which in turn improves the computational cost. To this end, the cranial surface is densely evaluated with a preselected number of points to represent anatomical regions of interest, P.

**Fig. 4:**
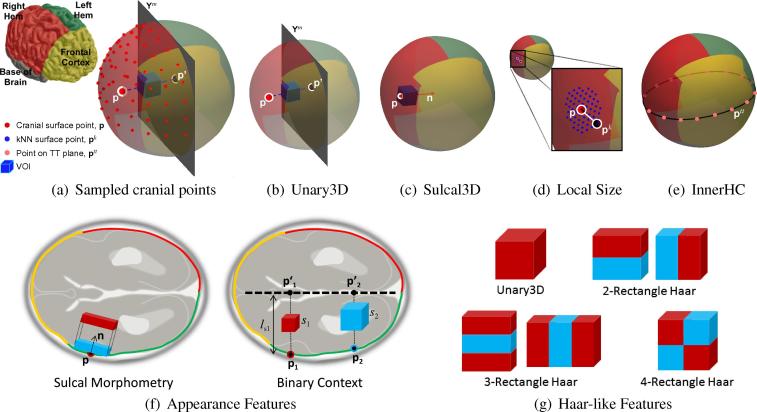
Feature design. Schematic representation of feature sets with respect to the parametrized cranial surface model. (a) Features are extracted by first sampling a cranial point p from a set of available surface points. A cuboidal VOI can be used to characterize appearance based on (b) Unary3D or (c) Sulcal3D features sets. Size features characterize (d) local skull deformations or (e) global head growth. (f) Appearance-based features are designed to capture sulcal changes and to compare structural changes between different brain regions. (g) Haar-like features are computed from the cuboidal VOI’s by subtracting the voxels within the red cuboid from those in the blue cuboid. (For interpretation of the references to colour in this figure legend, the reader is referred to the web version of this article.)

However, due to the effects of cranial thickening, the brain hemisphere proximal to the US probe is typically occluded, leaving only the distal hemisphere with visible and discernible intracranial structures. As such, feature extraction is confined to points on half of the cranial surface $P^{h}\in R^{3\times N_{P^{h}}}$ corresponding to the distal cerebral hemisphere in the image volume, such that $h\in \left\{L\text{,}R\right\}$ denotes the hemisphere in question (Fig. 4(a)). For simplicity, the surface is divided by the midsagittal plane $Y^{m}\in R^{3\times 2}$ and the sparse subset of points $P^{h}$ is a matrix of $N_{P^{h}}<N_{U}$ (Fig. 4(a)).

*Appearance-based features*. This bank of features extracts information from the greyscale US image voxels in order to capture age-related sonographic patterns of an anatomical region. Given the points on the deformed surface $P^{h}$ and the 3D US image, a cuboidal volume-of-interest (VOI) is sampled based on the cranial points available on the selected hemispheric subsurface (Fig. 4(a)). The location of the VOI is determined by the position of the sampled cranial point p, and its normal vector n.

VOI dimensions are defined by a scalar side length, *s*, which is scaled with respect to distance between cranial point p and its projection onto the midsagittal plane, $p^{\prime }$, such that $s=l_{s}\|p-p^{\prime }\|$, where $l_{s}∼U(0\text{,}0.5)$ is randomly selected during feature evaluation. Scaling allows for characterization of local anatomy independent of cranial surface size such that like-for-like anatomical comparisons can be achieved. The exclusion of age-related brain/cranial growth factors allowed by relative VOI sizing is paramount to retaining the ‘pure appearance characterisation’ quality of this feature set.

Upon extraction of the image voxels within the cuboidal VOI, the feature score is evaluated by computing one of the Haar-like features shown in Fig. 4(g). In particular, the score is determined by subtracting the sum of voxels in a cuboid from the sum of voxels in an adjacent cuboid of the same dimensions (shown as red and blue cuboids in Fig. 4(g)). The cuboids are of arbitrary dimensions and aspect ratio, and they are sensitive to edges and ridge-like structures within the image.

Appearance-based features comprise of two groups: sulcal and intracranial VOIs. Sulcal features are evaluated by affixing the VOI to the inner cranial surface (Fig. 4(c)). They are designed to capture the sonographic image appearance related to changes in shape and morphology of the sulci and gyri on the cortical surface across gestation. When evaluating sulcal features, the VOI is oriented along the normal vector n at cranial point p (Fig. 4(f)).

Intracranial features, on the other hand, are evaluated by displacing the VOI along the vector normal to $Y^{m}$ (Fig. 4(b)). The VOI displacement $d_{\mathrm{VOI}}=r\|p-p^{\prime }\|$ is determined in proportion to the distance between p and $p^{\prime }$, where r∼U(0,1) allowing for the VOI to be placed anywhere in the trajectory between the inner skull and the falx cerebri (or midsagittal plane, $Y^{m}$), ultimately covering the entire brain space. The value of *r* is also randomly selected during feature evaluation. This relative VOI positioning encourages correspondence in the sampling of intracranial anatomical regions regardless of brain size.

*Local size features*. These features capture local skull deformations and hence local growth patterns at different time points in pregnancy. To compute this feature, a cranial point p is first sampled from the set of available points on the cranial subsurface of interest. Using a Ball tree search algorithm (Omohundro, 1989), *k* cranial points nearest to p are identified (k=9). During feature evaluation, one of the *k*-th nearest neighbours, $p^{k}$, is randomly selected and the Euclidean or orthogonal distance between the sampled cranial point (Fig. 4(d), in red) and its randomly-selected *k*-th nearest neighbour (Fig. 4(d), in black) is obtained in Euclidean space $R^{3}$:

(6) $$d_{\mathrm{Euclid}}=\|p-p^{k}\|$$

and the orthogonal size features are the x,y,z-component distances given by:

(7) $$d_{⊥}^{j}=\left|p_{j}-p_{j}^{k}\right|\text{,}\;\text{where}\;j=\left\{1\text{,}2\text{,}3\right\}$$

*Biometric features*. Guided by current clinical assessment of fetal growth, the biometric feature is akin to the clinical head circumference (HC) measurement acquired from the standard transthalamic (TT) plane of the head (ISUOG, 2007). In this case, the feature is evaluated as the perimeter of the inner contour of the deformed cranial surface at the level of the diagnostic TT plane, $Y^{\mathrm{tt}}$. To define a parametric representation of the TT plane on the cranial surface, the plane is first identified on a single reference brain volume by manual selection of three cranial points, $Z^{\mathrm{tt}}\in R^{3\times 3}$. The surface points defining the TT plane, $P^{\mathrm{tt}}$, are the surface points closest to $Z^{\mathrm{tt}}$.

At feature evaluation, the inner cranial contour (or inner HC) is obtained from each image by extracting the 2D TT plane $Y^{\mathrm{tt}}$ defined by $P^{\mathrm{tt}}$ and computing the closed path length of all the surface edges which the plane intersects. The biometric HC feature captures global changes in head size in a manner similar to the current clinical method of GA estimation (Namburete et al., 2014a), emulating rigid cranial transformations related to fetal growth.

### 2.3 Modelling age-specific sonographic appearance

Our goal is to use age-discriminating image information to predict GA from 3D images of the brain. To develop the predictive age model, we take advantage of the regression forest framework that is well-established in the literature (Breiman, 2001; Criminisi et al., 2012) to produce estimates of continuous variables. Given that GA is itself a continuous variable, we opted for training the forest from a large longitudinal dataset of brain US images spanning the entire age range of interest, detailed in Section 2.3.1. The process of predictive age regression is explained in Section 2.3.2.

#### 2.3.1 Learning the link between age and brain images

The model is trained on a supervised dataset, in which all training examples are annotated with the GAs at scanning. In our framework, each training example $V_{i}=(I_{i}\text{,}P_{i}^{h}\text{,}a_{i})$ comprises of a 3D US image $I_{i}$ and its corresponding deformed cranial surface parametrization $P_{i}^{h}$, labelled with the GA of the fetus at the time of scanning, $a_{i}$. The dataset is denoted by $V=\{V_{i}\}$.

We train our trees following the regression forest framework proposed by Criminisi et al. (2012). During training, a random subset of the dataset $V^{t}\subset V$ is traversed through each tree $T_{t}$ in the regression forest $F=\{T_{t}\}$. All training examples in the subset are passed into the root node $\left(V_{j=0}^{t}\right)$ of each tree, and the data is recursively partitioned at each node *j* in its path along the tree branches until reaching leaf nodes. At each node, the data is split to the left and right child nodes, $V_{j\in \{L\text{,}R\}}^{t}$, by age-discriminating binary tests according to the node optimization criteria described below. Data splitting continues until leaf nodes are created due to (a) a maximum number of tree levels (i.e. tree depth, *d*) has been reached; (b) the node contains less than a defined number of training data points and (c) a minimum information gain has been achieved.

*Node optimization*. The age-discriminating binary tests are designed to minimize the differential entropy between the data sent to the left and right children nodes. At each node, a feature $f_{i}$ is randomly-selected from the available feature sets $f\in N^{n}$ (described in §2.2.1). The feature is applied to the dataset at the node $V_{j}^{t}\subset V^{t}$ and data splitting between children nodes occurs on the basis of a threshold, *τ*. The optimally age-discriminating node feature is determined by applying *m* features (m≪n) to $V_{j}^{t}$ to identify the pair of $\left(f_{i}\text{,}\tau \right)$ which minimizes the cost function.

Modelling the ages of the dataset at each node *j* as a random variable $a_{j}$ with univariate Gaussian distribution, $a_{j}∼N({\overset{¯}{a}}_{j}\text{,}\sigma_{j}^{2})$, the cost function can be expressed as

(8) $$I_{g}=\log\left|\sigma_{j}^{2}(V_{j}^{t})\right|-\underset{i\in \left\{L\text{,}R\right\}}{\sum }\omega_{i}\log\left|\sigma_{i}^{2}\left(V_{j}^{t\text{,}i}\right)\right|$$

where ${\overset{¯}{a}}_{j}$ is the mean age of all samples at the *j*-node, $\sigma^{2}$ denotes the variance of the ages, and $\omega_{i}$ is the ratio between the number of training examples in a child node $V_{j}^{t\text{,}i}$ and the number of examples in the parent node $V_{j}^{t}$, i.e. $\omega_{i}=\left|V_{j}^{t\text{,}i}\right|/\left|V_{j}^{t}\right|$. Maximizing Eq. 8 is equivalent to favouring binary tests which minimize the variance of the ages of the partitioned data, ultimately reducing the uncertainty in the age-discriminating ability of a given test on $V_{j}$. The discriminative quality of these binary tests hinges on the assumption that $V^{t}$ is representative of the complete dataset, V.

Each leaf node *l* stores the mean ${\overset{¯}{a}}_{l}$ and variance $\sigma_{l}^{2}$ of a Gaussian distribution derived from the vector of ages $a_{l}$ to have reached it:

(9) $$p_{l}(a)=N\left({\overset{¯}{a}}_{l}\text{,}\sigma_{l}^{2}\right)$$

#### 2.3.2 Prediction of GA

During age prediction, an unseen data point of unknown GA, $W_{k}=(I_{k}\text{,}P_{k}^{h})$, traverses through the nodes in each tree of the trained forest model, and the binary test associated with each node evaluates whether to send the data to the left or right child nodes, until $W_{k}$ eventually reaches a leaf node. For each tree, the leaf node reached provides a mean age estimate with an associated variance. Leaf nodes with high variance values have lower age certainty, so they are assumed to be less informative and likely to add noise. Therefore, a single prediction $a_{k}$ is generated by taking the mean of only the age estimates with associated variance less than $\sigma_{\max}^{2}$. Specifically:

(10) $$A=\left\{a_{l}|\sigma_{l}^{2}<\sigma_{\max}^{2}\right\}\text{,}\;a_{k}=\frac{1}{\left|A\right|}\underset{a_{l}\in A}{\sum }a_{l}$$

where $\left|A\right|$ is the number of ages satisfying the exclusion criteria. For the experiments that follow $\sigma_{\max}^{2}=1.0\;{\text{GW}}^{2}$.

## 3 Dataset and implementation details

### 3.1 US data and preprocessing

The model was developed to characterize all brain regions observable in the distal hemisphere of US images. The input data to the framework are 3D US images of the fetal brain obtained from two study databases: (a) INTERGROWTH-21st,^3^ an optimally healthy group of women with a low risk of pregnancy complications and fetal abnormalities (Papageorghiou et al., 2014) (Dataset A); and (b) INTERBIO-21st,^4^ an unselected, routine clinical cohort (Dataset B). Throughout the text, we refer to these as Dataset A and Dataset B, respectively. ‘True age’ (${\mathrm{GA}}_{\mathrm{true}}$) was defined by the last menstrual period (LMP) and confirmed by crown-rump-length (CRL) measurement on US images taken in the first-trimester ($⩽14^{+0}$ weeks) agreeing within 7 days. For both datasets, CRL-based age is accurate within 2.7 days, determined from 3 independent clinical measurements. For this work, the age prediction model was trained using 448 3D US images of the fetal brain (198 and 250 images with visible left and right hemispheres, respectively) acquired from Dataset A ranging from $18^{+0}\;\text{to}\;33^{+6}$ GW. Cross validation experiments were conducted on the same dataset following a leave-10-out protocol. The image inclusion criteria were:

– Cranium occupies ⩾50% of the image.
– Distal cerebral hemisphere is not occluded.
– Interhemispheric fissure is clearly visible in the entire supratentorial region.
– Intracranial structures (namely, Sylvian fissure, thalami, ventricles, and cavum septum pellucidum) are clearly visible.

3D US images of the fetal head were collected using a Philips HD9 curvilinear probe at a 2–5 MHz wave frequency. All images used for training and validation of the model were preprocessed by first resampling the acquired images to an isotropic spatial resolution of $0.6\times 0.6\times 0.6\;{\text{mm}}^{3}$. Ridge-like structures were then enhanced using a bandpass Gaussian derivative filter (kernel size $▽G_{\sigma }=4\;\text{mm}$). Cranial parametrization was then applied to the preprocessed data as detailed in Section 2.1. The bandpassed 3D US images and their respective deformed parametric surfaces were passed into the regression forest algorithm for training and testing.

### 3.2 Parameters and training

Regression forests consist of several parameters which can be set based on the desired application. For the task of neurosonography-based GA estimation, each regression forest was constructed by training T=20 trees to a maximum depth of $d_{\max}=15$. At each node *j*, a total of m=200 features was sampled, from which only one feature $f_{j}$ was selected as possessing the most age-discriminating power. The age regression framework was implemented in C++ (3.30 GHz quad-core, 12 GB RAM), and (as expected) training time proved to be related to the size of the training dataset (data not shown). For each tree, training took an average of **4.6** **min** and **7.2** **min** for left and right distal hemispheres, respectively, and approximately **0.1** **s** to predict GA from a single volume.

### 3.3 Evaluation of GA predictions

To measure the accuracy of GA predictions, we used the root-mean-squared error (RMSE):

(11) $$\mathrm{RMSE}=\sqrt{\frac{1}{\left|W\right|}\sum_{k=1}^{\left|W\right|}{\left(a_{k}^{\prime }-a_{k}\right)}^{2}}$$

where $\left|W\right|$ is the number of test images, $a_{k}^{\prime }$ is the model-predicted GA of the *k*-th test image, and $a_{k}$ is ${\mathrm{GA}}_{\mathrm{true}}$.

## 4 Results

In the following, we describe experiments conducted in order to demonstrate the performance and functionality of the age regression forest for the task of estimating GA from 3D US images of the fetal brain.

### 4.1 Model parameter and feature selection

**Fig. 5:**
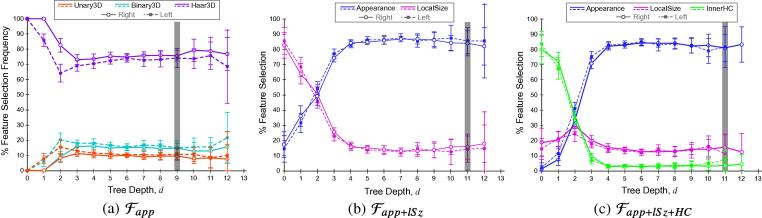
Feature selection profiles. Feature selection versus tree depth for the leave-10-out cross validation forests (a) $F_{\mathrm{app}}$, (b) $F_{\mathrm{app}+\mathrm{lSz}}$, and (c) $F_{\mathrm{app}+\mathrm{lSz}+\mathrm{HC}}$. Lines denote mean feature selection frequency at a given tree depth (solid: right distal hemisphere; dashed: left distal hemisphere). Error bars indicate standard deviation. Grey vertical lines indicate the tree depth of optimal GA prediction.

To gain insight into the functionality of the GA prediction model, we assessed the relative importance of each feature set presented in Section 2.2.1 in its age discriminating ability. This was achieved by plotting forest level (*d*) against the number of times a particular feature $f_{i}$ was selected at each level in the forest, normalized by the total number of nodes present at the given level across all trees in the forest ($N_{d}$), i.e. $\frac{N_{d}(f_{j})}{N_{d}}$. This result is shown in Fig. 5 and it is based on the understanding that decision trees select more general binary tests to split the data at the shallower levels and progressively select more specific tests as the data traverses to deeper levels. In our work, such an analysis informs on which features contain global GA discriminating ability, and which provide information about subtler or more detailed age-related differences. Three separate forests were trained for comparison (Table 1).

Fig. 5 demonstrates the feature selection profiles for forests (a) $F_{\mathrm{app}}$, (b) $F_{\mathrm{app}+\mathrm{lSz}}$, and (c) $F_{\mathrm{app}+\mathrm{lSz}+\mathrm{HC}}$ for the leave-10-out cross-validation forests, displaying each appearance and size feature subset separately. It is evident from Fig. 5(a) that when the forest is trained exclusively with appearance-based features ($F_{\mathrm{app}}$), Haar-like features are selected more frequently than Unary or Binary context features. At all forest levels, Haar3D was consistently selected in more than 60% of the nodes as the most powerful feature. This indicates that the algorithm selected binary tests which capture ridge-like image features more frequently than those capturing differences in homogeneous image regions, suggesting that sulcal and fissural development was salient in the brain during the second and third trimester. Fig. 5(b) shows that when the feature vector comprises of appearance and local size features, the latter are more age-discriminating in the shallower forest levels, but anatomical appearance is dominant from level d=2, and optimal prediction with minimal RMSE occurs at level d=11. However, when the innerHC feature is also included in the feature vector (Fig. 5(c)), it is preferentially selected in the shallower tree levels, superseding even local size features, before anatomical appearance features are rendered more important also at level d=2. This clearly demonstrates that global and local head size provide an important first estimate of GA, but sonographic anatomical appearance is valuable in refining age predictions.

To assess the stability of feature selection, cross-validation experiments were conducted using a leave-10-out protocol, each time partitioning the dataset such that 10 images were kept for testing the model, whilst the remaining images (i.e. 188 and 240 images with visible left and right distal hemispheres, respectively) were used for training the corresponding regression forests. Fig. 5 displays error bars indicating the standard deviation of feature selection frequency for all forests trained during cross-validation. The fact that the error bars tightly follow the curves for all feature profiles demonstrates that the model is stable in its selection of age-discriminating features at the different forest levels. It is also clear from the figure that regardless of which cerebral hemisphere was used to train the model, the feature selection profiles were similar. This demonstrates the ability of the model to simply capture anatomical progression of the brain, respecting cerebral developmental symmetry.

**Fig. 6:**
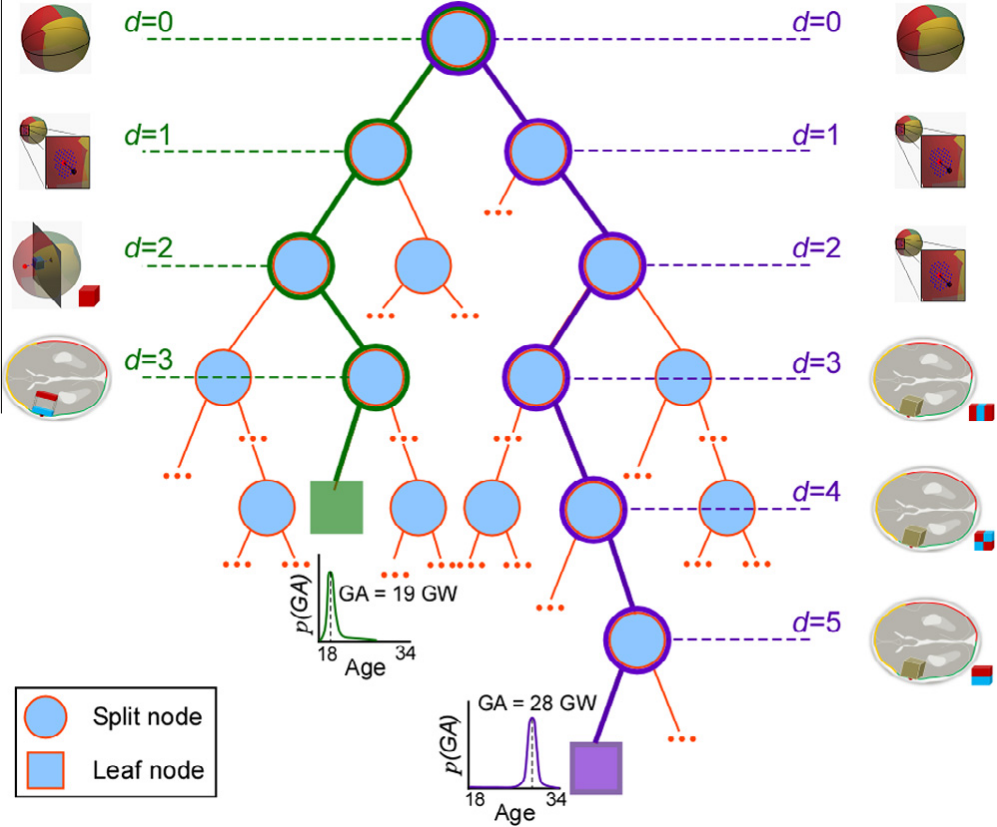
Example feature selection. Illustration of feature selection paths for two different fetuses at 19 and 28 GW, demonstrating typical tree traversal.

**Fig. 7:**
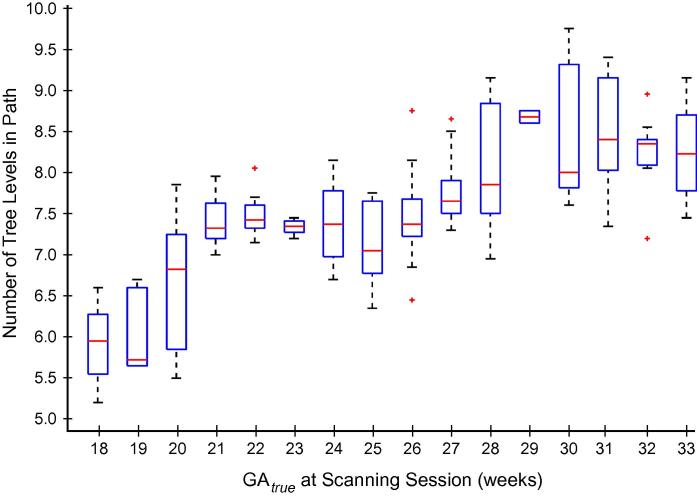
Tree traversal path length versus GA. Box plot of the average number of tree levels in the decision forest path (from root to leaf) for the test examples at each GA. Median is denoted by a red line in each box, and the edges indicate the 25th and 75th percentiles. Outliers are plotted individually. (For interpretation of the references to colour in this figure legend, the reader is referred to the web version of this article.)

Fig. 6 illustrates the process of feature selection and demonstrates the tree traversal paths for two data examples from fetuses at $19^{+3}$ and $28^{+3}$ GW for a typical tree from forest $F_{\mathrm{app}+\mathrm{lSz}+\mathrm{HC}}$. Note that although the examples traverse the tree along different paths before reaching a leaf node, the binary tests applied to achieve GA estimation follow a similar pattern. Specifically, the inner HC is tested at the root node (d=0), followed by local size features in the shallow levels (d=1-2), and lastly image appearance features were applied as the final tests before arriving at a leaf node where a decision about GA is achieved. This corroborates the findings of the feature selection profiles plotted in Fig. 5, and most test examples followed a similar tree traversal pattern. Also, the example from the younger fetus had a shorter tree traversal path than the older fetus. Guided by this indication, we found that on average, the trend was for the tree traversal path length to increase with GA (Fig. 7), which may be indicative of the longitudinal behaviour of the model as the brain increases in complexity.

### 4.2 Brain maturation maps

Having established the method by assessing the entire image volume of each distal hemisphere, it is expected that only some regions within the entire 3D cerebral volume would accurately report on brain maturation, while low-contrast or highly variable regions could obscure this task. We hypothesized that the unguided training framework could by itself identify the relevant brain regions that provide the best age discrimination. Similar to the works of Sabuncu and Van Leemput (2012), Toews et al. (2010) and Konukoglu et al. (2013) on MR brain images, we examined the most age-discriminating brain regions selected by the algorithm. To achieve this, heat maps were generated to demonstrate the image appearance features selected in each level of a forest. To generate these maps, the nodes in each forest level *d* were parsed, and the image voxels pertaining to the VOI selected at a node were incremented each time they were sampled. The resulting image was then normalized by the number of nodes present at level *d*, i.e. $N_{d}$.

**Fig. 8:**
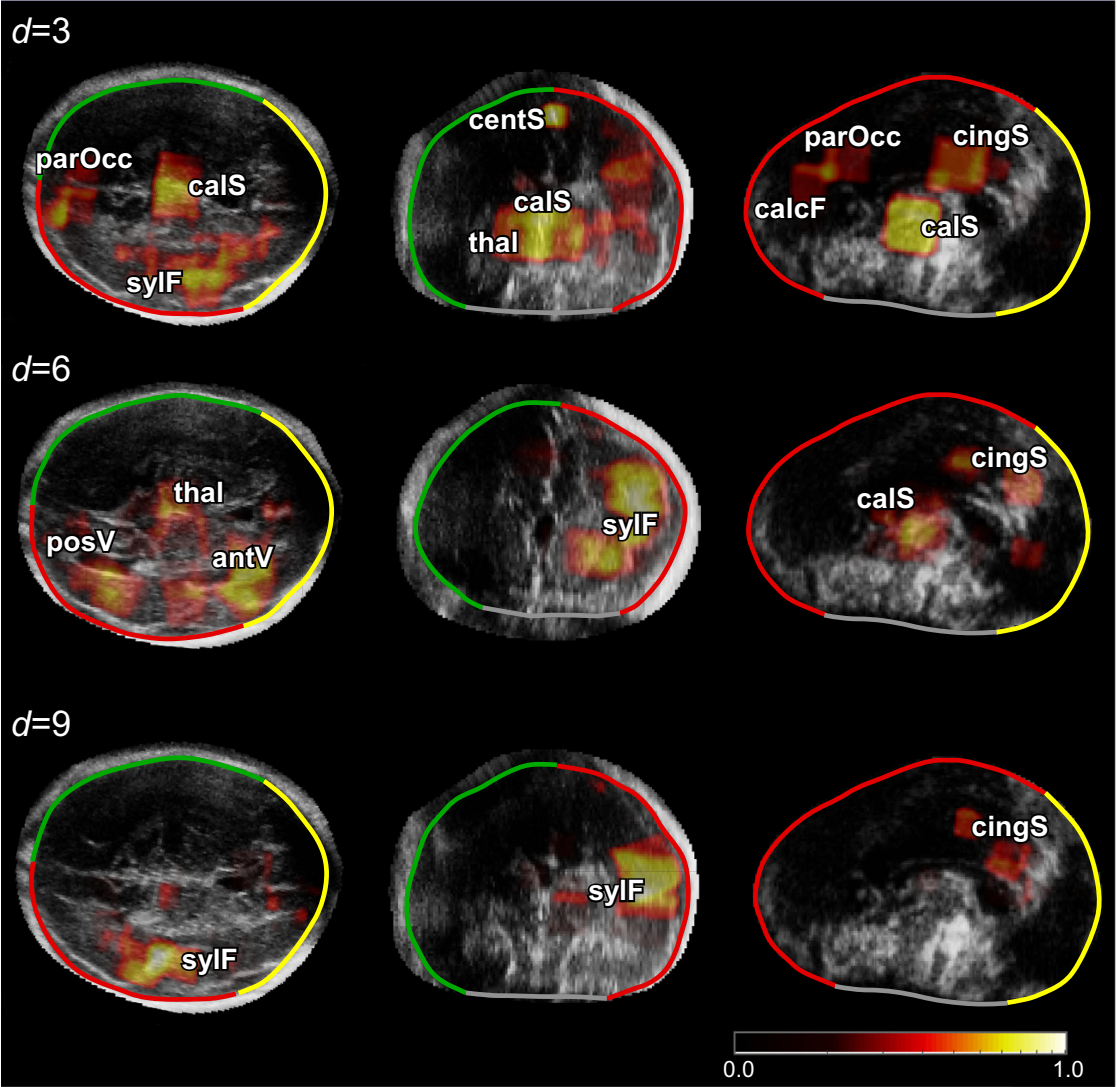
GA-discriminating brain regions. Visual example of the feature locations selected at three different levels of the forest (d=3,6,9) superimposed on axial (left column), coronal (centre column), and sagittal slices (right column). The heat map corresponds to the relative feature importance, such that bright regions correspond to frequently selected brain regions. *sylF*: Sylvian Fissure, *calS*: Callossal Sulcus, *centS*: Central Sulcus, *parOcc*: Parieto-occipital Fissure, *cingS*: Cingulate Sulcus, *calcF*: Calcarine Fissure, *posV*: Posterior Ventricle, *antV*: Anterior Ventricle, *thal*: Thalami.

Fig. 8 demonstrates the locations of age-discriminating image features selected by the model at three different levels of the forest. Our investigation showed that the algorithm consistently selected a small number of key age-discriminating regions. These included: (*a*) regions around the midsagittal plane such as the callossal sulcus, thalami, and parieto-occipital fissure at d=3; (*b*) the posterior and anterior ventricles at d=6; and (*c*) sulcal areas such as the Sylvian fissure (d=3,6,9) and central sulcus (d=3). These are regions which show substantial change during gestation (Toi et al., 2004; Pistorius et al., 2010), reflecting the ability of the model to query developmentally-informative anatomical regions during the GA prediction process. We also observed that at shallow forest levels, the model queried more brain regions than at deeper levels. For instance, at d=3 the model queried six key regions (i.e. callossal sulcus, thalami, parieto-occipital fissure, central sulcus, cingulate sulcus, and Sylvian fissure), while it focused primarily on the Sylvian fissure and cingulate sulcus at d=9. Given that the nodes at deeper levels have lower variance values, it indicates that the binary tests associated with these nodes improve the confidence of GA estimates, highlighting finer differences between age-related sonographic appearance. In theory, it might be expected that the regions queried by these nodes would be spatially distributed in the brain. However, in practice, we found that key anatomical regions were consistently selected by different trees, identifying them as salient landmarks for GA estimation.

### 4.3 Model-based prediction of age

**Fig. 9:**
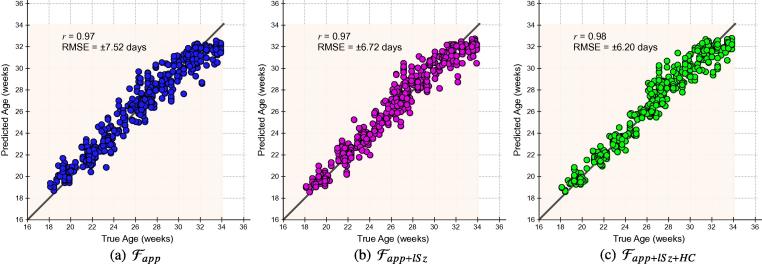
Regression results from cross-validation experiments. Graphs plot true age versus predicted age for each image in the training database (Dataset A) using a leave-10-out protocol. Shown are the age predictions achieved using forests trained with different feature sets: from left to right, (a) $F_{\mathrm{app}}$, (b) $F_{\mathrm{app}+\mathrm{lSz}}$, (c) $F_{\mathrm{app}+\mathrm{lSz}+\mathrm{HC}}$. The *r* value and root-mean-squared error (RMSE) are provided for each plot. The rectangular overlay indicates the age range of data included in this work (i.e. $18^{+0}\text{to}33^{+6}$ GW).

**Fig. 10:**
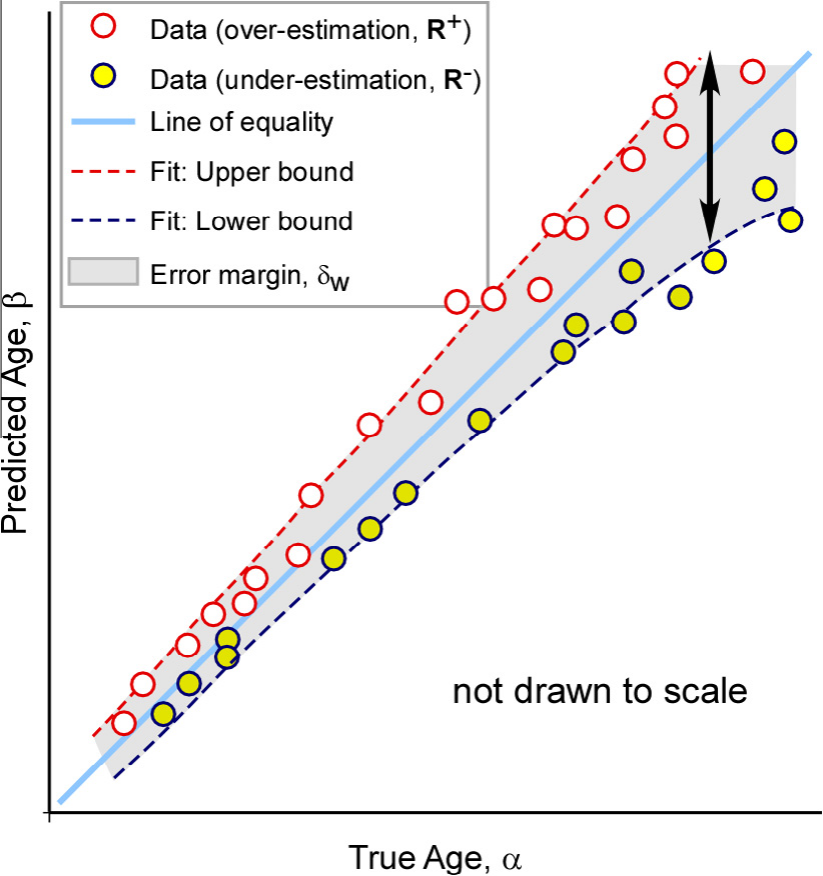
Error margin of the GA predictions, $\delta_{w}$. Illustration of the $\omega_{\mathrm{CI}}$. The positive and negative GA predictions (relative to the line of equality) are marked as red and yellow circles, respectively. Quadratic fitting functions approximating the positive ($\zeta^{+}$, dashed red line) and negative ($\zeta^{-}$, dashed blue line) bounds of the confidence interval are shown, and the difference between these functions denotes the value at $\delta_{w}$ when sampled at each age value (grey region). (For interpretation of the references to colour in this figure legend, the reader is referred to the web version of this article.)

**Fig. 11:**
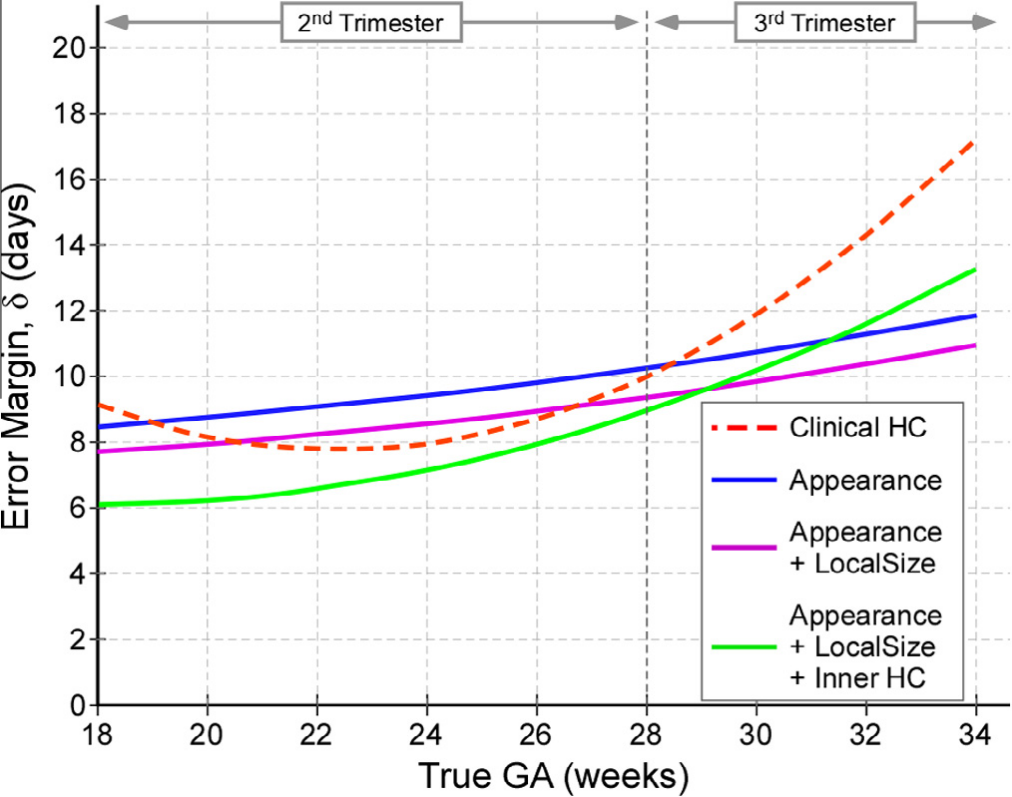
Model comparison. Error margin of GA prediction, $\delta_{w}$, expressed in days, for the best current clinical method (dashed red line), and for each trained forest model (solid lines) during the second and third trimesters of pregnancy. (Results shown for Dataset A.)

To demonstrate the predictive quality of each combination of feature sets across gestation and hence identify the best predictor, we plotted the age predictions from the leave-10-out cross-validation experiments on Dataset A (Fig. 9). Our results show that the forest which combines all available features ($F_{\mathrm{app}+\mathrm{lSz}+\mathrm{HC}}$) yields the most accurate age predictions. However, even the forest trained exclusively with anatomical appearance features ($F_{\mathrm{app}}$) was capable of generating predictions with a high *r*-value (r=0.97) and low RMSE. We also plotted the curves denoting the the error margin of the GA predictions ($\delta_{w}$) against GA, shown in Fig. 11. The different types of forests were applied to Dataset A and each was compared to ${\mathrm{GA}}_{\mathrm{true}}$ on the basis of $\delta_{w}$. Although the forests were set to train to a maximum depth of $d_{\max}=15$, none of the trees reached this depth during training, and optimal results were achieved at d=10 for $F_{\mathrm{app}}$, and d=12 for both $F_{\mathrm{app}+\mathrm{lSz}}$ and $F_{\mathrm{app}+\mathrm{lSz}+\mathrm{HC}}$. To compute the $\delta_{w}$, the GA predictions (with respect to the line of equality) were separated into two groups: over-estimations made by the model $R^{+}\in R^{2\times N_{+}}$, and the under-estimations $R^{-}\in R^{2\times N_{-}}$ in GA prediction. Quadratic fit functions were generated to model each of the positive ($\zeta^{+}$) and negative ($\zeta^{-}$) bounds of the GA predictions. The $\delta_{w}$ for each GA prediction model was defined as the absolute difference between the fit functions for the upper and lower centiles, $\delta_{w}=|\zeta^{+}-\zeta^{-}|$. We illustrate this procedure in refer to Fig. 10.

A good age prediction model would demonstrate low $\delta_{w}$ values, indicating a narrow error range (i.e. narrow spread). According to Fig. 11, all predictors produce the lowest errors in GA estimation during the second trimester, and error increases steadily with GA. However, the rate of increase differs for the predictors. For instance, the $\delta_{w}$ of both $F_{\mathrm{app}}$ and $F_{\mathrm{app}+\mathrm{lSz}}$ increases almost linearly at a rate of +0.226days/GW and +0.215days/GW, respectively. The width of the clinical HC predictions shows a sharp quadratic increase such that the error nearly doubles from $18^{+0}$ to $33^{+6}$ GW, rendering the GA predictions in the third trimester ≈10 days more erroneous. On the other hand, the forest containing all features $\left(F_{\mathrm{app}+\mathrm{lSz}+\mathrm{HC}}\right)$ produces the lowest errors in the second trimester, but mimics the behaviour of clinical HC predictions in the third trimester.

We also observed that model-based predictions improved with each feature set that was incorporated into the feature vector. Specifically, at 18 GW, $F_{\mathrm{app}}$ yields the widest error margin ($\delta_{w}=8.2$ days) whilst $F_{\mathrm{app}+\mathrm{lSz}+\mathrm{HC}}$ has the narrowest ($\delta_{w}=6.2$ days). Although this is not strictly the case in the third trimester, $F_{\mathrm{app}+\mathrm{lSz}}$ continues to yield lower GA errors than $F_{\mathrm{app}}$. Nevertheless, this experiment identifies forest $F_{\mathrm{app}+\mathrm{lSz}+\mathrm{HC}}$ as yielding the most accurate age estimates overall, and hence the highest predictive power of the GA estimation model. In addition, the fact that the error margins of model-based predictors were always narrower than that of clinical HC demonstrates the ability of the model to outperform the best current clinical method for the entire GA range considered in this work.

### 4.4 Estimation and validation of age predictions

**Fig. 12:**
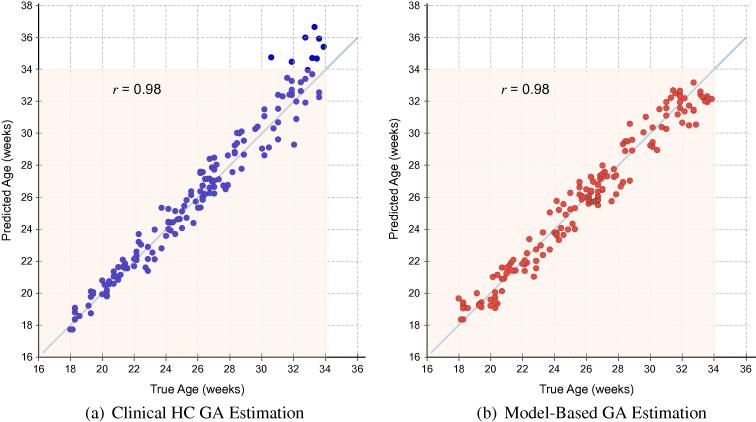
GA regression results. GA estimation results for a routine clinical dataset, using (a) the best current clinical method (blue), and (b) the $F_{\mathrm{app}+\mathrm{lSz}+\mathrm{HC}}$ forest model (red). The rectangular overlay indicates the age range of data included in this work (i.e. $18^{+0}\;\text{to}\;33^{+6}$ GW). (For interpretation of the references to colour in this figure legend, the reader is referred to the web version of this article.)

Model-based age predictions (${\mathrm{GA}}_{\mathrm{model}}$) were compared against the best clinical method for estimating gestational age, ${\mathrm{GA}}_{\mathrm{clinical}}$: the mean of three HC measurements taken from independent 2D US images of the TT plane and regressed to population growth charts (Loughna et al., 2009). Table 2 summarizes the GA estimation results from applying $F_{\mathrm{app}+\mathrm{lSz}+\mathrm{HC}}$ and the clinical HC method to Dataset B, and Fig. 12 plots the GA predictions against true GA. As a comparison, a predictor using random guessing would result in a RMS error of ±2.76GW(≈19.4days). Our results demonstrate that although ${\mathrm{GA}}_{\mathrm{clinical}}$ yields the lowest prediction errors in the second trimester (7.2 days), ${\mathrm{GA}}_{\mathrm{model}}$ has a lower overall error throughout the entire age range, outperforming ${\mathrm{GA}}_{\mathrm{clinical}}$ by ±0.91 days and reducing the CI by ±1.68 days. However, the real benefit of ${\mathrm{GA}}_{\mathrm{model}}$ becomes apparent in the third trimester where the CI is reduced by approximately ±4.56days and GA predictions are improved by ±2.51days. Furthermore, visual inspection of Fig. 12 indicates that the results from ${\mathrm{GA}}_{\mathrm{model}}$ had a tighter fit to the line of equality for the entire age range, whereas results from ${\mathrm{GA}}_{\mathrm{clinical}}$ diverged with progressing GA.

### 4.5 Developmental trajectories

**Fig. 13:**
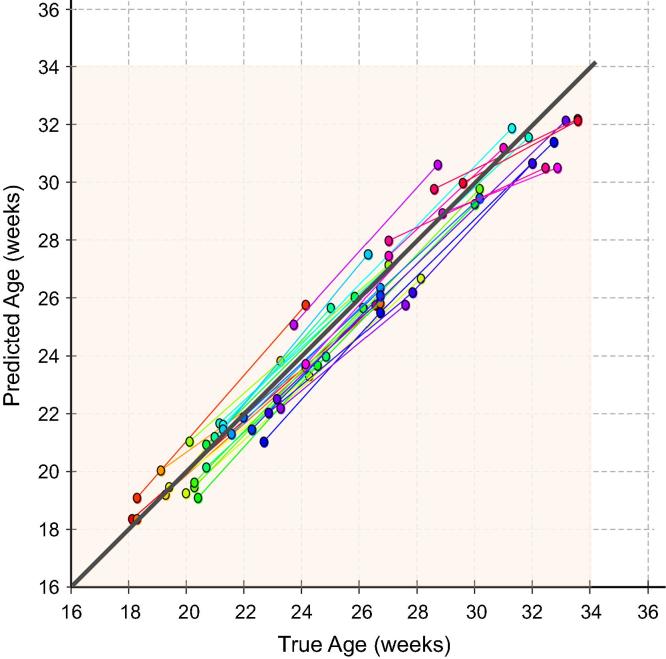
Developmental trajectories. Longitudinal age predictions of images taken from fetuses at multiple separate scanning sessions. Connected line segments correspond to a single patient, and each individual scanning session is denoted by a circle.

In order to determine whether the model produces chronologically consistent age predictions between scanning session, we assessed the developmental trajectories for a subset of fetuses scanned at multiple time points in pregnancy. Fig. 13 compares the GA trajectories as predicted by separate scanning sessions for 31 subjects from Dataset B. The lines denote the time lapses between subsequent scans (marked by circles), and connected circles correspond to a single patient. It is evident that there is high agreement between the true age at a scanning session, and the model-predicted GA. This demonstrates the ability of the model to consistently approximate GA with monotonically consistent time lapses between the predictions of each scanning session. This is also indicative of the potential for using the model to extract personalized maturational progression across different time points in pregnancy.

## 5 Discussion

For the task of GA estimation from US scans of the fetal brain, this paper presents and validates a semi-automated learning-based framework for discovering age-related sonographic patterns in the images and linking them to neurodevelopmental maturation. Our model benefits from a surface manifold representation of the fetal skull which allows for fast and efficient sampling of anatomically-corresponding brain regions to achieve like-for-like structural comparison of different developmental stages and serves as a skull-stripping tool. We demonstrated that the model is capable of characterizing neuroanatomical appearance both spatially and temporally, modelling GA as a continuous variable from $18^{+0}\;\text{to}\;33^{+6}$ GW, capturing the natural variation existing in a healthy fetal population over an age range of active brain maturation.

Clinically relevant metadata (i.e. fetal head circumference) was provided as input to the machine learning framework, and in addition, we extended canonical features sets (e.g. Haar-like features (Viola and Jones, 2004)) to capture structural changes within the fetal brain. Our results indicate that features which inform on cranial (and hence brain) size are selected as the first tests for discriminating between age groups, but structural image appearance features inform of finer age-related differences. This suggests that the method that is currently used in the clinic provides a good first prediction of GA, but additional information about structural brain development reduces errors in GA estimation and improves the confidence of predictions throughout the entire age range.

Validation of the model revealed that (*a*) the model will generalize to an independent dataset and (*b*) that feature sets are consistently selected by the different forests, hence indicating that the model represents a stable solution for GA estimation. It is worth noting that due to the visualization of only one cerebral hemisphere in every given brain US image, each forest was trained *separately* to be applied to its respective hemisphere. That is, if only the left cerebral hemisphere is clearly discernible in a test image, age prediction will be obtained by applying the forest trained using only left-hemisphere images in order to ensure developmental and anatomical hemispheric likeness between training and testing data. We found that regardless of which cerebral hemisphere is observable in the US image and thus used for training the regression forest, an accurate age prediction can be achieved using our model, reflecting its ability to capture developmental symmetry.

Our model demonstrated the feasibility of automatically learning the pattern of sonographic activity and linking it to GA. In particular, the model was able to identify relevant brain regions such as the Sylvian fissure, cingulate sulcus, and callosal sulcus as powerful image regions in the age-discrimination task. This corroborates findings in the clinical literature about these cerebral structures following a characteristic pattern of development (Toi et al., 2004; Mittal et al., 2007; Pistorius et al., 2010). Beyond the extraction of anatomical regions which undergo significant changes during gestation, our GA estimation framework has the potential to provide clinically relevant information.

Finally, our GA estimation framework has the potential to provide clinically relevant information. On dataset B, the presented algorithm improved the confidence of age predictions provided by the clinical HC method by ±0.64 days and ±4.57 days in the second and third trimesters, respectively. Specifically, our third trimester predictions within ±7.77 days are a notable improvement on the ±18 days reported in the literature (Hadlock et al., 1983). Moreover, the fact that model-based prediction errors increased with GA may be reflective of the inherent biological variation as gestation progresses, ultimately making the task of GA estimation more challenging. In fact, the largest errors were seen in the third trimester when the model relied more on clinical HC than on image appearance. This behaviour may be attributed to progressive skull thickening which results in increasing structural occlusions, reducing the image-based support available to the GA estimation task. This, in conjunction with the fact that decision tree traversal path lengths increased with GA, demonstrate the longitudinal behaviour of the model as it is able to respond to the fact that GA estimation is more complex in later gestation.

In conclusion, we have developed a novel feature-based model which regresses brain development to GA using US images, which has never before been attempted. Validation on a healthy dataset of 448 fetuses demonstrated the ability of the model to accurately approximate true chronological age. Application of the framework to a routine clinical fetal cohort of 187 fetuses resulted in GA prediction accuracy of ±6.1 days, particularly outperforming the current clinical method of GA estimation in the third trimester. Our preliminary analysis identified age-discriminating brain regions observable in fetal US images, namely the Sylvian fissure, callosal sulcus, and parieto-occipital fissure, all of which have been reported as undergoing significant change during the gestational period.

## Figures and Tables

**Table 1:** Description of the different types of regression forests trained for model selection, and their constituent feature sets. Graphical explanation of each feature set is provided in Fig. 4 and discussed in Section 2.2.1.

Forest type	Feature vector	
$F_{\mathrm{app}}$	Appearance only	
$F_{\mathrm{app}+\mathrm{lSz}}$	Appearance and local size	
$F_{\mathrm{app}+\mathrm{lSz}+\mathrm{HC}}$	Appearance, local size, and biometry	

**Table 2:** GA prediction performance measures for the clinical HC method and the best model-based predictor ($F_{\mathrm{app}+\mathrm{lSz}+\mathrm{HC}}$) applied to Dataset B for the 2nd and 3rd trimesters of pregnancy. RMSE and confidence interval (CI) values expressed in days.

Trimester	2nd		3rd		2nd and 3rd	
Age range, weeks	$18^{+0}\text{"–"}27^{+6}$		$28^{+0}\text{"–"}33^{+6}$		$18^{+0}\text{"–"}33^{+6}$	
Age mean (SD), weeks	23.45 (2.94)		31.07 (1.76)		25.83 (4.41)	
No. subjects, *n*	108		49		157	

Performance measure	RMSE (*r*)	CI	RMSE (*r*)	CI	RMSE (*r*)	CI

Clinical HC prediction	4.86 (0.94)	±9.46	10.28 (0.76)	±18.57	7.01 (0.98)	±13.32
Model-based prediction	5.18 (0.97)	±10.10	7.77 (0.83)	±14.01	6.10 (0.98)	±11.64

## Footnotes

1 http://www.vtk.org.

2 Standard “Snakes” fixes U.

3 http://www.intergrowth21.org.uk.

4 http://www.interbio21.org.uk.
